# Case study: persistent recovery of hand movement and tactile sensation in peripheral nerve injury using targeted transcutaneous spinal cord stimulation

**DOI:** 10.3389/fnins.2023.1210544

**Published:** 2023-07-17

**Authors:** Santosh Chandrasekaran, Nikunj A. Bhagat, Richard Ramdeo, Sadegh Ebrahimi, Pawan D. Sharma, Doug G. Griffin, Adam Stein, Susan J. Harkema, Chad E. Bouton

**Affiliations:** ^1^Neural Bypass and Brain Computer Interface Laboratory, Institute of Bioelectronic Medicine, Feinstein Institutes for Medical Research, Northwell Health, Manhasset, NY, United States; ^2^Department of Physical Medicine and Rehabilitation, University of Texas Health Science Center, Houston, TX, United States; ^3^Kentucky Spinal Cord Injury Research Center, University of Louisville, Louisville, KY, United States; ^4^Northwell Health STARS Rehabilitation, East Meadow, NY, United States; ^5^Department of Physical Medicine and Rehabilitation, Donald and Barbara Zucker School of Medicine at Hofstra, Northwell Health, Manhasset, NY, United States; ^6^Department of Bioengineering, University of Louisville, Louisville, KY, United States; ^7^Frazier Rehabilitation Institute, University of Louisville Health, Louisville, KY, United States; ^8^Department of Neurological Surgery, University of Louisville, Louisville, KY, United States; ^9^Donald and Barbara Zucker School of Medicine at Hofstra/Northwell, Manhasset, NY, United States

**Keywords:** peripheral nerve injury (PNI), spinal cord stimulation (SCS), neuromodulation, motor restoration, tactile sensation

## Abstract

Peripheral nerve injury can lead to chronic pain, paralysis, and loss of sensation, severely affecting quality of life. Spinal cord stimulation has been used in the clinic to provide pain relief arising from peripheral nerve injuries, however, its ability to restore function after peripheral nerve injury have not been explored. Neuromodulation of the spinal cord through transcutaneous spinal cord stimulation (tSCS), when paired with activity-based training, has shown promising results towards restoring volitional limb control in people with spinal cord injury. We show, for the first time, the effectiveness of targeted tSCS in restoring strength (407% increase from 1.79 ± 1.24 N to up to 7.3 ± 0.93 N) and significantly increasing hand dexterity in an individual with paralysis due to a peripheral nerve injury (PNI). Furthermore, this is the first study to document a persisting 3-point improvement during clinical assessment of tactile sensation in peripheral injury after receiving 6 weeks of tSCS. Lastly, the motor and sensory gains persisted for several months after stimulation was received, suggesting tSCS may lead to long-lasting benefits, even in PNI. Non-invasive spinal cord stimulation shows tremendous promise as a safe and effective therapeutic approach with broad applications in functional recovery after debilitating injuries.

## Introduction

Peripheral nerve injury (PNI) can lead to uncomfortable sensations such as tingling, paresthesia, and chronic pain. Depending on the severity of the injury, PNI can also result in loss of somatosensation, fine motor control and dexterity, and even paralysis, thereby drastically impacting quality of life. Neuromodulation targeted at the spinal cord through electrical stimulation has been routinely used in the clinic to mitigate chronic pain, including that arising from PNIs (Gupta et al., 2020). Traditionally, physical therapy has been recommended to aid recovery in cases of PNI-induced paralysis. However, the benefits can often be limited in terms of recovered range of motions and/or strength (Maugeri et al., 2021). Thus, there is a major need to develop novel techniques for restoring lost motor and sensory capacities as a result of PNI.

Spinal cord stimulation has shown great promise in restoring voluntary control of muscles of the upper-limb in spinal cord injury (Freyvert et al., 2018; Gad et al., 2018; Inanici et al., 2021) and stroke (Powell et al., 2023). Pharmacological and computational work (Capogrosso et al., 2013) suggest that activation of the dorsal fibers via spinal cord stimulation increases the excitability of the local spinal circuitry including the efferent fibers. Pairing transcutaneous spinal cord stimulation (tSCS) at the cervical levels with intense motor training can result in sustained improvements in hand and arm function (Benavides et al., 2020; Zhang et al., 2020; Inanici et al., 2021).

We hypothesized that activating the local spinal circuitry through tSCS could help decrease the effects of peripheral nerve injury. In this pilot study, we performed targeted transcutaneous stimulation of the cervical spinal cord paired with specific activity-based training in one individual with a peripheral nerve injury. We used a custom electronically-configurable electrode array to target specific cervical levels. This enabled us to choose the precise location of stimulation to achieve maximal recruitment of the muscle group of interest. Even though the participant received stimulation and motor training only once per week, we observed a rapid increase in both volitionally controlled muscle activity, effective force and increase in somatosensation within a period of 5–6 weeks.

Taken together, this study demonstrates the advantages of targeted tSCS paired with activity-based training in restoring volitional control of upper-limb movement and improvements in tactile sensitivity in case of peripheral injury.

## Methods

### Participant

A 48-year-old woman presented with partial paralysis of the left hand due to peripheral nerve injury leading to chronic median and ulnar nerve neuropathy. She was involved in a multi-vehicle accident in 2019. Two days later, she noticed an impairment in her ability to move her left thumb and other fingers limiting her gripping and functional activities. It was assessed that the absence of left thumb movement appeared to be neurological in nature possibly originating from neck or thoracic outlet. She reported neck pain with left radicular symptoms, decreased range of movement in her cervical spine. An electrodiagnostic study conducted in July 2020 observed no evidence of cervical radiculopathy. Both studies repeatedly found median and ulnar nerve neuropathy at the wrist.

At the onset of this study, the participant had severe atrophy of the left abductor pollicis brevis (APB) muscle with no motor units. The median motor nerve was nonreactive at the wrist, and severe ulnar neuropathy at the elbow. The participant was enrolled into this current study in December 2020. The clinical assessment scores taken pre-intervention, at the end of intervention and at follow-up sessions are provided in Supplementary Table S1. All study procedures were approved by the Northwell Health Institutional Review Board. The study was conducted in accordance with the principles embodied in the Declaration of Helsinki and in accordance with local statutory requirements. The participant provided formal written informed consent to participate in this study. The study has been registered with ClinicalTrials.gov (NCT04755699, first posted on 16/02/2021).

### Intervention protocol

#### Before intervention period

Baseline clinical assessments, namely the Graded Redefined Assessment of Strength, Sensation and Prehension (GRASSP), were performed before the onset of any intervention. These assessments were repeated for two sessions, scheduled a month apart, to establish a pre-intervention baseline.

#### Start of the intervention period

At the beginning of the intervention period, we characterized the tSCS-mediated muscle recruitment profile for the participant. tSCS consisted of a 10 kHz biphasic sinusoidal waveform with a pulse duration of 1 ms. Low frequency stimulation (3 Hz) of increasing current amplitudes was sequentially targeted at different locations along the rostrocaudal axis of the cervical region and the resultant compound action potentials from specific muscles of the arm and hand were recorded. Based on the specificity of tSCS in recruiting the different motoneuron pools, we choose the specific location where we subsequently localized the tSCS to be paired with the activity-based training.

#### Intervention period

After the pre-intervention baselines were established, we began the intervention period wherein the study participant attended 4-h long experimental sessions in the lab once per week. To evaluate the therapeutic effects of tSCS, we focused on specific muscles. At the beginning of every session, we measured both the evoked force and electromyographic (EMG) activity in these target muscles without any stimulation occurring simultaneously. Following that, the participant received transcutaneous spinal cord stimulation (tSCS) in the cervical region for 1 hour each session. During tSCS, the participant was administered activity-based training which involved performing tasks specifically designed to activate the target muscles. The intervention period in this manuscript took place over 35 weeks.

#### ‘No Stim’ period

After 16 weeks of receiving tSCS, we administered a 3-week period when the participant received no stimulation, but continued to perform the tasks that constituted their activity-based training. Weekly administration of tSCS was resumed after this ‘No Stim’ period.

#### End of intervention period

tSCS was resumed after the *No Stim* period up to the end of 35 weeks since the beginning of intervention. Force and EMG were recorded as before.

### EMG signal processing

Bipolar surface EMG electrodes were used to record muscle activity from the following muscles of the left arm and hand: biceps brachii (BIC), triceps brachii (TRI), flexor digitorum superficialis (FDS), extensor digitorum communis (EDC) and abductor pollicis brevis (APB). We chose these muscles as they are primarily innervated by the cervical roots, namely BIC: C5, TRI: C7, FDS: C8, EDC: C7, ABP: C7. We used pre-gelled Ag/AgCl electrodes (Ø 24 mm, Arbo™ H124SG, Covidien), connected to EMG sensors (AT-04-001 MyoWare™ Muscle Sensor), a differential amplifier (AD6221) with a 2nd order bandpass filter (20-498 Hz), and a signal digitizer (PicoScope® Model 4824A). The MyoWare sensor required the electrodes to have a center-to-center distance of 30 mm. The sampling rate used was 10 MHz while characterizing the recruitment profile of the upper limb muscles and 10–20 kHz during task performance. For characterizing the area-under-the curve (AUC), the EMG signal was digitally filtered using a 60 Hz IIR comb filter and a 6th-order Butterworth bandpass filter between 10–1,000 Hz in MATLAB.

### Transcutaneous spinal cord stimulation (tSCS)

**Figure 1:**
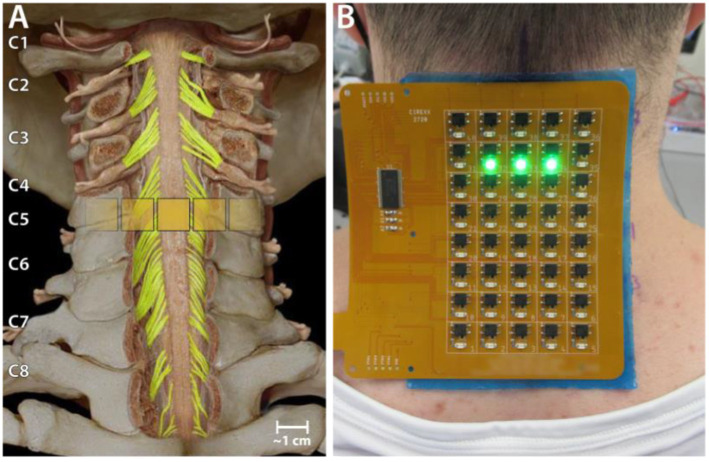
Experiment setup. **(A)** Schematic showing the location of a 1 × 3 activated electrode configuration superimposed over the human spinal cord showing the dorsal column and roots. **(B)** The custom electronically configurable electrode array placed over the cervical spinal cord of a study participant with a 1 × 3 configuration of activated electrodes (green LEDs).

Transcutaneous spinal cord stimulation was provided using a custom-built stimulator and electrode array. The stimulator consisted of a microcontroller to produce the stimulation waveform digitally, a Texas Instruments class-D amplifier (TAS5825P) in voltage mode, and a 12:1 step-up transformer (Xicon 42TM003-RC). The stimulator was powered by a 24 V battery and a safety circuitry was used to restrict power density levels to no more than 0.25 W/cm^2^. The flexible PCB electrode array consisted of electroless nickel immersion gold (ENIG) or immersion silver-plated square contacts (10 mm x 10 mm) arranged in an 8 × 5 pattern with a 11 mm center-to-center electrode separation. Electrical stimulation to any combination of the 40 electrodes within the array, was controlled using solid-state relays mounted above each of the electrodes and a custom MATLAB-based GUI. Miniature green LEDs mounted on the dorsal surface of the electrode array, visually indicated the active electrodes. To target specific cervical segments (Figure 1A), an electrode configuration of 1 × 3 (Figure 1B) was used wherein stimulation was provided simultaneously to 3 adjacent electrodes within a single row, spanning the midline. The electrode array was affixed to the back of the neck using a rectangular piece of proprietary hydrogel. To ensure consistency in placement of the array between sessions, we used the inion of the external occipital protuberance as a landmark. Distances measured from the inion were used to place the electrode array and identify the location of stimulation. Two interconnected 5 × 10 cm rectangular self-adhesive hydrogel electrodes (Axelgaard Manufacturing Co., Ltd., United States) placed along the midline over the lumbar spinal cord served as return electrodes (anodes). Stimulation consisted of a 10 kHz biphasic sinusoidal waveform with pulse duration of 1 ms delivered at 3 Hz for generating recruitment profiles.

Stimulation frequency was set to 50 Hz when tSCS was paired with activity-based training and the pulse width was reduced to 0.5 ms to reduce neck muscle activation and increase participant comfort. Stimulation amplitude was gradually raised up to the maximal level that the participants found tolerable while not interfering with their training, which was usually 140–160 mA. tSCS paired with activity-based training was administered for 45–60 min per session.

### Experimental sessions

Heart rate and blood pressure were measured at the beginning and end of each session. For finer measurements of voluntary muscle control, we measured both the evoked force and electromyographic (EMG) activity in the muscles of the affected hand and arm (left).

The tasks involved activating the D1 interphalangeal joint and separately generating maximal force while performing a lateral pinch grasp. For this, a custom rig was used. The participant was asked to push down on the sensor (load range of 10 lb., FX29 compact compression load cell, TE Connectivity) using their thumb. For all force measuring tasks, the experimenter cued the participants to push against the force sensor for 3–5 s and then to relax for 3–5 s. The task was performed in 2 sets of 5 trials each with a 60–90 s of rest period between the sets. Verbal encouragement was provided to encourage the participants to generate maximal force and to sustain (if possible) for the full cued period. The sensors used were calibrated at regular intervals using standard weights (Supplementary Figure S1).

While receiving tSCS, the participant performed the same tasks as described above. This served the dual purpose of providing activity-based training while receiving tSCS as well as measuring the volitional EMG activity and force.

### tSCS-mediated muscle recruitment profile

Stimulation consisted of a 1 ms-long multiphasic pulse of the 10 kHz sinusoidal waveform at a stimulation frequency of 3 Hz. Stimulation amplitudes tested ranged from 100 mA to up to 225 mA in intervals of approximately 25 mA. This was repeated for each of the eight rows of electrodes on the array. The Picoscope 6 acquisition software (Pico Technology, Cambridgeshire, United Kingdom) was used to trigger acquisition of a 100 ms-long EMG signal following each stimulation pulse. For each stimulation amplitude and at each electrode row, we recorded such an EMG signal from 20–30 repetitions of the stimulation pulse. All the data was imported into MATLAB for further analysis. From each of the EMG signals recorded, we isolated a snippet starting from 5 ms and ending at 55 ms after the stimulation artifact. We measured the peak-to-peak amplitude (P2P) for this snippet. It was included for analysis only if the maximum amplitude of the snippet was greater than 5 times the standard deviation of baseline signal of that recording channel. For each muscle, the P2P amplitudes were normalized to the maximal P2P amplitude recorded across all amplitudes and electrode rows.

X-ray images for the participant in the sagittal plane with radio-opaque markers on the neck were obtained to determine actual location of electrodes with respect to spinal roots and vertebral landmarks.

We also measured the stimulation current of maximal activation for each muscle. For this, at each cervical level, we increased the amplitude until the reflexive activation of the muscle was visually determined to be consistent for every stimulation pulse and did not change for any small increase in amplitude. This stimulation amplitude was noted as the Maximal Activation Current for that muscle at the cervical level. This procedure was repeated for each muscle.

### Functional outcome

In addition to the areas recommended in the GRASSP assessment for sensibility evaluation, we tested additional relevant spots on the volar aspect of the hand to obtain a comprehensive profiling of the sensory changes in the entire hand. Specifically, we included the remaining finger pads and four corners of the palm. To provide encouragement and also characterize the true capability of the participant, we let the GRASSP prehension assessment tasks exceed the stipulated time of 75 s.

### Statistical analysis

For statistical analysis of the evoked force, we compared the forces recorded in three stages of the study (start, no-stim and end of study). For the early stage, we chose the first three consecutive sessions after the start of weekly tSCS sessions. For the no-stim stage, we chose three sessions during the period when tSCS was not being delivered. For the end of study stage, we chose the last three consecutive sessions. For the force measurements involving the D1 IP muscle in case of the participant with the peripheral nerve injury, we performed a *t*-test. We employed the Bonferroni correction for all multiple comparisons.

## Results

Our results demonstrate that transcutaneous spinal cord stimulation can be used to activate specific motoneuron pools in the cervical spinal cord. Moreover, targeted tSCS paired with minimal activity-based training resulted in a substantial and sustained increase in muscle activity and strength in specific upper-limb muscles.

### Recruitment of upper limb muscles through targeted tSCS

**Figure 2:**
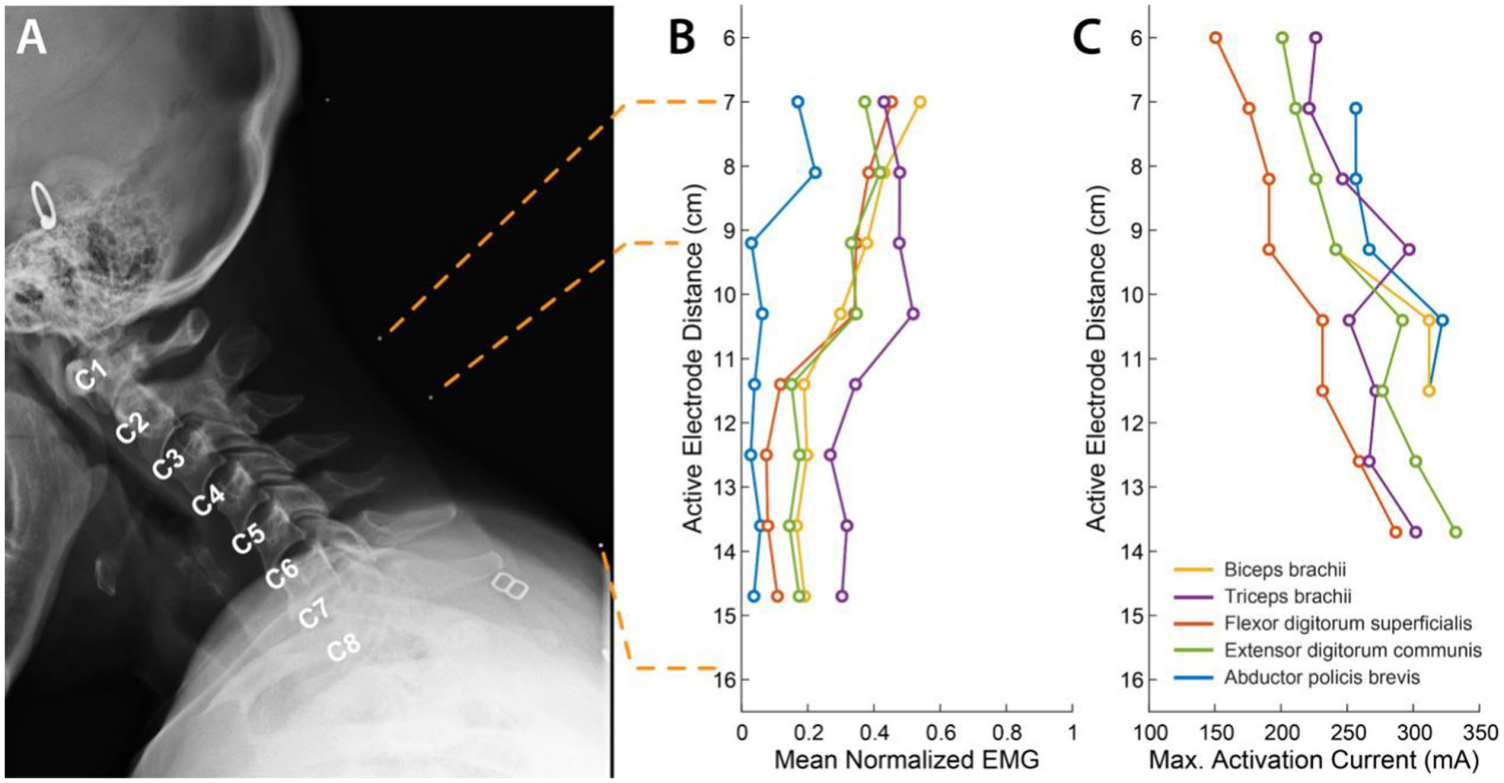
Muscle recruitment profile during cervical tSCS. **(A)** X-ray images in the sagittal plane with radio-opaque markers on the neck (white dots). The topmost marker identifies the inion of the external occipital protuberance. The second and third markers identify points 7 cm and 9.2 cm respectively, from the inion signifying the first and third rows of a putative electrode array whose first row of electrodes was aligned at 7 cm from the inion. The last marker identifies the location of the last row of the putative electrode array at 15.7 cm from the inion. The cervical labels mark the exit point of the respective dorsal roots. **(B)** Mean activation of the 5 muscles across all stimulation amplitudes mediated by tSCS through each of the 8 electrode rows. **(C)** Stimulation amplitude that resulted in maximal activat In review ion of each of the 5 muscles.

We characterized the recruitment profile of the different motor pools innervating the upper limb muscles. To determine the effect of the location of stimulation along the rostro-caudal axis on the recruitment of the upper limb muscles, we sequentially delivered stimulation to different electrode triplets in the array (Figure 2A). Simultaneously, we recorded EMG activity from 5 muscles of the left arm and hand. We hypothesized that the recruitment pattern through transcutaneous stimulation would reflect the rostrocaudal segment-wise distribution of the upper limb motor nuclei in the cervical spinal cord. Almost all the muscles exhibited the best recruitment when stimulation was delivered around the C5-C6 level. Stimulating using rostral electrodes primarily activated the biceps (Figure 2B). Activation of the triceps invariably was strongest when stimulation was localized around the C5-C6 level. Stimulation using electrodes positioned more caudally resulted in decreased recruitment across all muscles. In participant CTS01, APB showed only weak recruitment irrespective of the location of stimulation, likely due to neuropathy. But we observed that APB was maximally recruited when stimulation was targeted at the C4-C5 level. Additionally, we also determined the current required for maximal activation (Max. Activation Current) for each of the upper limb muscles. The thresholds of activation showed similar results as the recruitment profiles with biceps and/or triceps muscles having a low threshold of activation during rostral stimulation (Figure 2C).

### Increased muscle activity and force generated with tSCS

We focused on the volitional control of the D1 interphalangeal (D1 IP) joint and lateral pinch. We chose the site of stimulation based on the choice of muscle and its activation profile. For the participant CTS01, we focused on thumb muscles, and delivered tSCS targeted at the C5 level (about 8 cm from the inion of the external occipital protuberance) based on the activation profile for the APB muscle.

**Figure 3:**
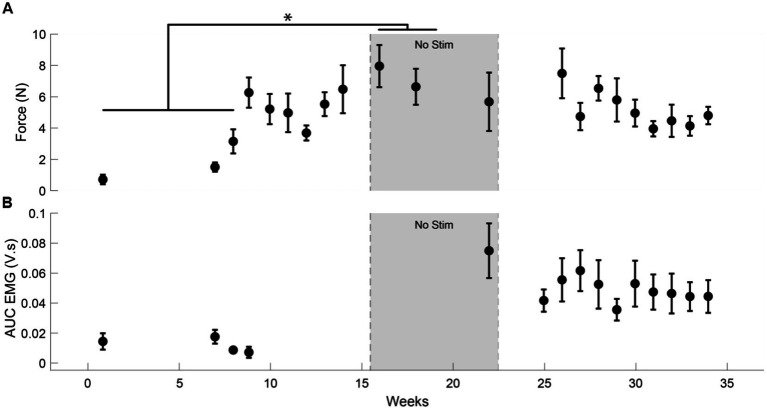
Increase in muscle activity and force after tSCS. **(A)** The force generated by the left D1 IP joint in case of participant CTS01. * indicates a significant difference (Two-sample t-test with Bonferroni correction for multiple comparisons). **(B)** The AUC of the EMG activity recorded from the left APB muscle during the task of force generation using the D1 IP joint.

We observed an average increase of 407% (*t*-statistic = −5.26, *p* < 0.05) in the force generated by the flexion of D1 IP joint (Figure 3A) in the ‘No Stim’ period (7.3 ± 0.93 N) at the end of 16 sessions compared to the forces at the start of the study (1.79 ± 1.24 N). This reduced to a 287% increase (5.15 ± 1.22 N) by the end of the study (30 sessions). This was accompanied by an increase in EMG activity of the left APB muscle (Figure 3B). We did not observe a significant increase in the lateral pinch force during this period.

### GRASSP assessments

**Figure 4:**
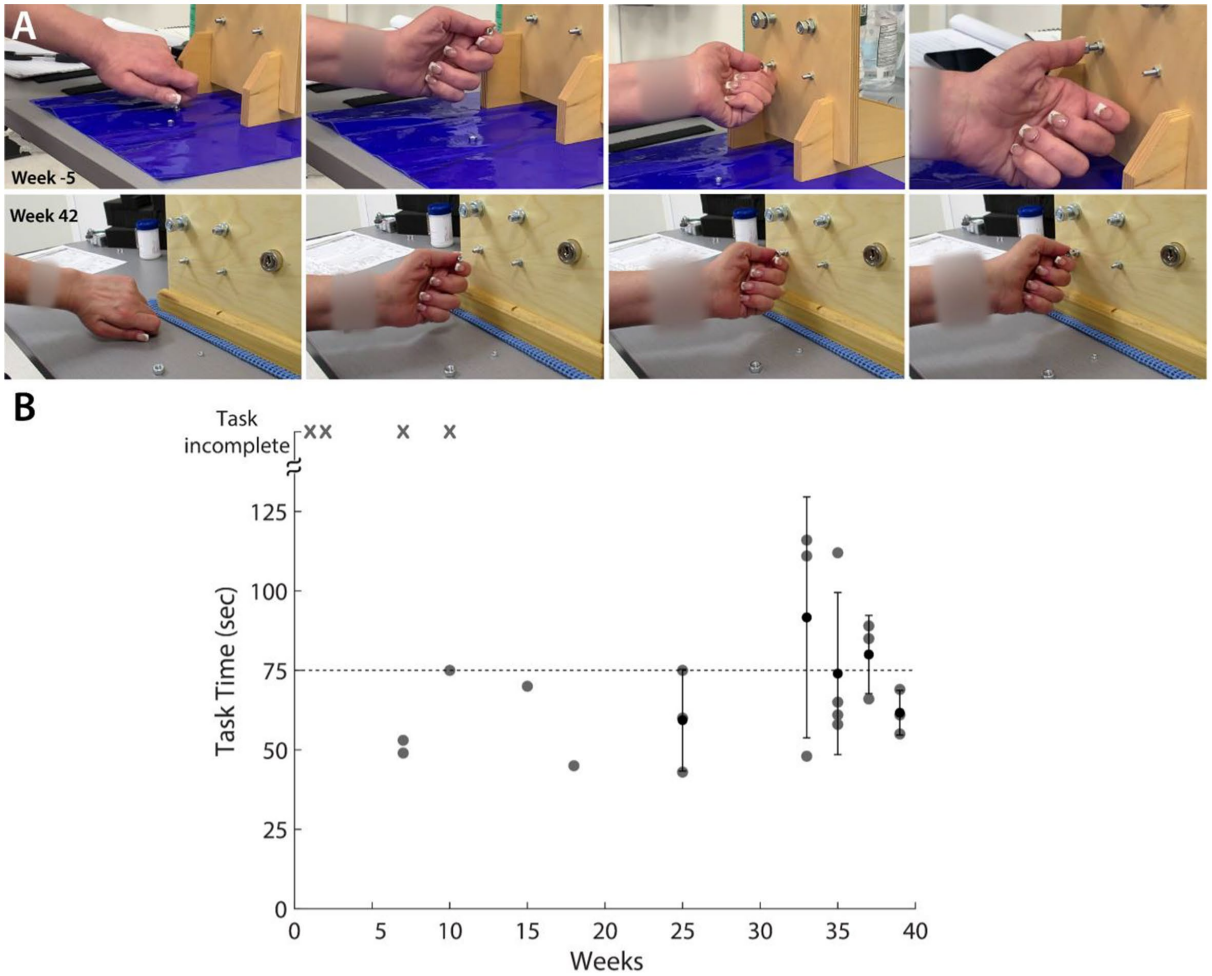
GRASSP Prehension Test – Nut threading. **(A)** Snapshots showing improvement in grasping behavior from before tSCS (first row) and week 42 of intervention (second row). **(B)** Scatter plot shows the improvement in the time required to perform the task. ‘x’ denotes that the participant could not complete the task.

To evaluate the clinical significance of the progress showed by the participant, we also performed standard clinical assessments, namely the GRASSP test. Most of the improvement was observed in the thumb as well as the ulnar side of the hand. GRASSP strength showed increases in D1 IP and D5 abduction movements (Supplementary Figure S2). GRASSP sensibility showed up to a 3-point increase in sensation at the pinky tip (Supplementary Figure S2). In the nut threading task included in the GRASSP prehension tests, the participant showed an improvement in performing the correct grasp (Figure 4A) and a decrease in the time taken to perform the task (Figure 4B). The participant also showed improvements in the range of movement of thumb abduction, flexion, and extension as well as index abduction (Supplementary Figure S3). A 1-year follow-up EMG and nerve conduction evaluation was administered. While the study stated that the sensory nerves remained unchanged, the motor study of both the median and ulnar nerves showed improvements.

## Discussion

In this study, we observed a substantial increase in volitionally generated force and EMG activity in muscles of the thumb, and significant increases in tactile sensation following administration of tSCS in an individual with a peripheral nerve injury. The gains in the both motor and sensory scores lasted up to 2 months even after stimulation had been withdrawn. Our design involved participants receiving spinal cord stimulation and activity-based training for only 1–2 h, once per week. We observed the changes in muscle force within 10 weeks of onset of tSCS. Importantly, our stimulation was targeted to achieve maximal recruitment of the muscle group of interest.

In addition to people with SCI, spinal cord stimulation (SCS) has recently shown promise in restoring movement in various other patient populations including stroke (Powell et al., 2023) and cerebral palsy (Hastings et al., 2022). These studies suggest that raising the excitability of the spinal cord neuronal networks could help alleviate the effect of abnormal synaptic connections resulting from a chronic neurological condition. The findings in this case study further suggest that spinal cord circuitry could be a key target for neuromodulation in case of peripheral neural injuries as well. SCS has shown modest success even in patients classified as motor-complete spinal cord injury. This is attributed to the presence of residual neural pathways that have been shown to persist even in cases of motor-complete SCIs (Sharma et al., 2023). It is possible that SCS could benefit a wide range of peripheral nerve injuries provided there is preservation of residual neural pathways.

The importance of activity-based training and the required dosage is certainly an unknown question. In our study, we establish that motor gains can be established with 8 weeks of receiving weekly tSCS. A similar timeline was established in our previous work in SCI and in a study with cerebral palsy (Hastings et al., 2022).Studies involving epidural stimulation of the spinal cord mediating restoration of voluntary motor control have shown the importance of targeted stimulation that can recruit specific motoneuron pools (Wagner et al., 2018; Rowald et al., 2022). A similar electrode configuration as the one used in this study has previously been demonstrated to be effective in recruiting specific motoneuron pools in able-bodied individuals (Gerasimenko et al., 2015; Krenn et al., 2015). In fact, lateralized stimulation shows greater selectivity in the motoneuron pools being activated (Oh et al., 2022; Bryson et al., 2023). In case of PNI, the recruitment of specific motoneuron pool that is most affected by the injury could be beneficial in terms of targeting the therapeutic benefits as well as increasing patient comfort.

One of the limitations of the study by nature of being a case study, is the possibility that the participant could have recovered from her condition spontaneously. However, in all the clinical assessments since the injury, it was evident that the participant was severely compromised in performing her activities of daily living and showed little improvement in the several years preceding the onset of stimulation. The rapid and objective improvements in her motor ability approximately 6 weeks after the commencement of tSCS (and years after injury) are highly unlikely to be solely due to spontaneous recovery.

Although the participant only performed a D1 IP and lateral pinch tasks during the activity-based training, the motor improvements helped her perform better at GRASSP prehension assessment subtest. This suggests that the activity-based training can help improve the volitional control of a group of muscles enabling increase in dexterity and grasp strength rather than being restricted to the target muscle/joint. Moreover, an increase from a starting D1 IP force of 1.8 N to up to 7.3 N may be modest, but is enough to recover functional use of the hand for the participant.

It was interesting to observe improvement in tactile sensation in the affected region for the participant. To our knowledge, this is the first study to document improvements in touch sensation in people with peripheral injury following tSCS. Stimulating dorsal roots of the spinal cord has been demonstrated to relay somatotopically relevant sensory information (Chandrasekaran et al., 2020). It may be worthwhile to explore the benefits of pairing tSCS with activities, such as tactile-oriented imagery, aimed at increasing somatosensory activation for the long-term rehabilitation of somatosensation in PNI.

In conclusion, our findings reported here suggest targeted transcutaneous spinal cord stimulation may have broader applications well beyond spinal cord or central nervous system injuries. Furthermore, with our previous work in developing brain-computer interface approaches for movement restoration based on decoding movement intention (Bouton et al., 2016, 2021), we believe that combining these technologies and approaches may lead to even better outcomes in traumatic injuries. By providing continual feedback to the user that is based on decoded motor activity representing movement intentions, user engagement may be increased and/or maintained. This feedback may help increase the strength of descending command signals sent from the motor cortex to the spinal cord. Decoded intentions can also be used to modulate tSCS patterns (temporally or spatially) in real-time to optimize EMG responses for the intended movement. This new approach may further expand the applications and implications of these powerful methods and deepen our understanding of how the human nervous system processes sensorimotor information.

## Data availability statement

The raw data supporting the conclusions of this article will be made available by the authors, without undue reservation.

## Ethics statement

The studies involving human participants were reviewed and approved by Northwell Health Institutional Review Board. The patients/participants provided their written informed consent to participate in this study.

## Author contributions

SC, NB, and CB designed the study. SC, NB, RR, SE, and CB performed the experiments. SC analyzed the data. NB designed and tested the electrode array, with guidance from CB. DG performed independent assessments of the motor and sensory capacity of the participants. PS and SH provided critical feedback and input. SC, NB, RR, SE, PS, DG, AS, SH, and CB contributed towards interpreting the results of the experiments. SC and CB finished the initial draft of the manuscript. All authors contributed to the article and approved the submitted version.

## Funding

This study was funded through support provided by Feinstein Institutes for Medical Research at Northwell Health.

## Conflict of interest

CB has multiple patents in related fields and financial interests in multiple start-up companies including Sanguistat, myString, and Neuvotion. SE has financial interests in Neuvotion, Inc.

The remaining authors declare that the research was conducted in the absence of any commercial or financial relationships that could be construed as a potential conflict of interest.

## Publisher’s note

All claims expressed in this article are solely those of the authors and do not necessarily represent those of their affiliated organizations, or those of the publisher, the editors and the reviewers. Any product that may be evaluated in this article, or claim that may be made by its manufacturer, is not guaranteed or endorsed by the publisher.
