# Supplementary material for: Case study: persistent recovery of hand movement and tactile sensation in peripheral nerve injury using targeted transcutaneous spinal cord stimulation
Source: Front Neurosci. 2023 Jul 17;17:1210544. doi: 10.3389/fnins.2023.1210544 (PMC10390294; doi:10.3389/fnins.2023.1210544)
Supplement: Supplementary file 1 [file Image_1.pdf]

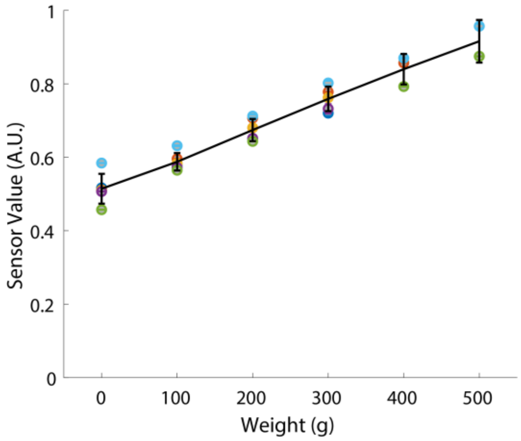

Supplementary Figure 1. Calibration curves for the sensor used in this study. Each color signifies a calibration test done on a different day. Black line signifies average calibration across days. Error bars shown are standard deviations.
