# Supplementary material for: Case study: persistent recovery of hand movement and tactile sensation in peripheral nerve injury using targeted transcutaneous spinal cord stimulation
Source: Front Neurosci. 2023 Jul 17;17:1210544. doi: 10.3389/fnins.2023.1210544 (PMC10390294; doi:10.3389/fnins.2023.1210544)
Supplement: Supplementary file 2 [file Image_2.pdf]

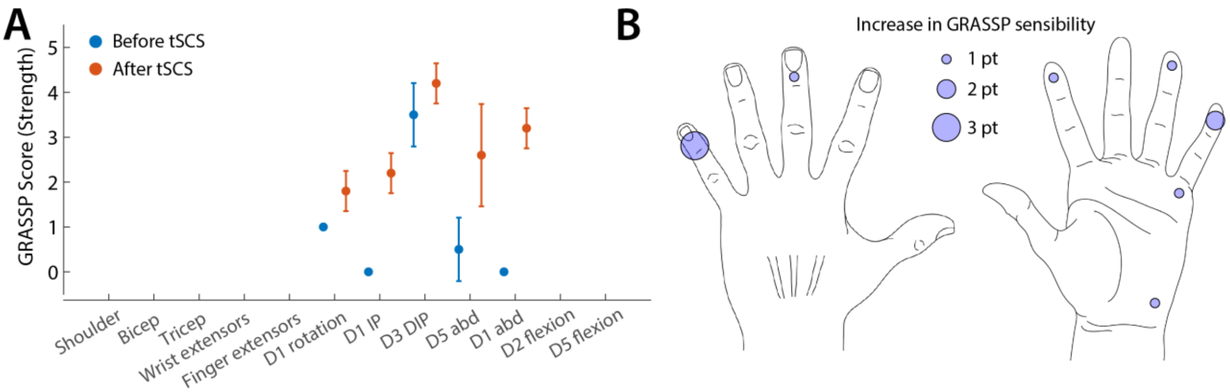

Supplementary Figure 2. GRASSP assessments. A) GRASSP strength change from baseline **B)** GRASSP sensibility change from baseline. Circles show 1- to 3-point increase in sensory perception as defined by the GRASSP sensibility scale.
