# Supplementary material for: Case study: persistent recovery of hand movement and tactile sensation in peripheral nerve injury using targeted transcutaneous spinal cord stimulation
Source: Front Neurosci. 2023 Jul 17;17:1210544. doi: 10.3389/fnins.2023.1210544 (PMC10390294; doi:10.3389/fnins.2023.1210544)
Supplement: Supplementary file 3 [file Image_3.pdf]

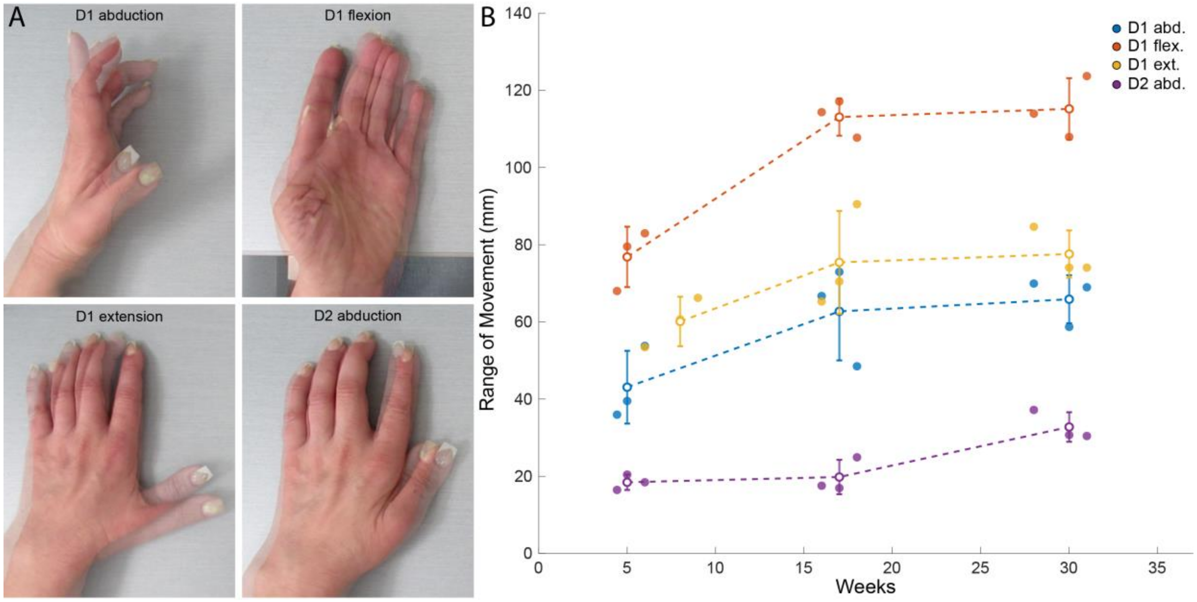

Supplementary Figure 3. Improvement in range of movement (ROM) in D1 abduction, D1 flexion, D1 extension and D2 abduction for the study participant. A) shows superimposed images from week 5 and week 31; B) Change in ROM for the 4 different movements.
