# Supplementary material for: Case study: persistent recovery of hand movement and tactile sensation in peripheral nerve injury using targeted transcutaneous spinal cord stimulation
Source: Front Neurosci. 2023 Jul 17;17:1210544. doi: 10.3389/fnins.2023.1210544 (PMC10390294; doi:10.3389/fnins.2023.1210544)
Supplement: Supplementary file 4 [file Table_1.pdf]

Supplementary Information

GRASSP Movement	Pre-Intervention Baseline 1		Pre-Intervention Baseline 2		At End of Intervention		1-month follow-up		2-month follow-up	
	**Left**	**Right**	**Left**	**Right**	**Left**	**Right**	**Left**	**Right**	**Left**	**Right**
**Shoulder**	5	5	5	5	5	5	5	5	5	5
**Biceps**	5	5	5	5	5	5	5	5	5	5
**Triceps**	5	5	5	5	5	5	5	5	5	5
**Wrist extensors**	5	5	5	5	5	5	5	5	5	5
**Finger extensors**	5	5	4	5	5	5	5	5	5	5
**D1 rotation**	1	5	1	5	2	5	2	5	2	5
**D1 IP**	0	5	0	5	3	5	2	5	3	5
**D3 DIP**	3	5	4	5	4	5	4	5	3	5
**D5 abduction**	1	5	0	5	2	5	4	5	3	5
**D1 abduction**	0	5	0	5	3	5	3	5	3	5
**D2 flexion**	3	5	4	5	3	5	3	5	3	5
**D5 flexion**	4	5	4	5	2	5	2	5	3	5
**GRASSP Sensation**										
	**Left**	**Right**	**Left**	**Right**	**Left**	**Right**	**Left**	**Right**	**Left**	**Right**
1 (D1 dorsum)		4	4	4	4	4	4	4	4	4
2 (D3 dorsum)		4	3	4	4	4	4	4	4	4
3 (D5 dorsum)		4	0	4	1	4	3	4	3	3
**Dorsal Total (12)**		**12**	**7**	**12**	**9**	**12**	**11**	**12**	**11**	**11**
4 (D1 palmar)	4	4	4	4	4	4	4	4	4	4
5 (D3 palmar)	4	4	4	4	4	4	4	4	4	4
6 (D5 palmar)	2	4	3	4	4	4	4	4	4	4
**Palmar Total (12)**	**10**	**12**	**11**	**12**	**12**	**12**	**12**	**12**	**12**	**12**
**Total (out of 24)**		**24**	**18**	**24**	**21**	**24**	**23**	**24**	**23**	**23**
Index	3	4	4	4	4	4	4	4	4	4
Ring	3	4	3	4	4	4	4	4	4	4
Palm (Index)	4	4	4	4	4	4	4	4	4	4
Palm (Pinky)	2	4	3	4	3	4	4	4	3	4
Palm (Base)	3	4	2	4	3	4	4	4	4	4
Palm (Thumb)	4	4	4	4	4	4	4	4	4	4

Supplementary Table 1. GRASSP movement and sensation scores
